# A literature review of biomarkers used for diagnosis of relative energy deficiency in sport

**DOI:** 10.3389/fspor.2024.1375740

**Published:** 2024-07-12

**Authors:** Kristýna Dvořáková, Ana Carolina Paludo, Adam Wagner, Dominik Puda, Marta Gimunová, Michal Kumstát

**Affiliations:** ^1^ Department of Sport Performance and Exercise Testing, Faculty of Sports Studies, Masaryk University, Brno, Czechia; ^2^ Department of Physical Activities and Health Sciences, Faculty of Sports Studies, Masaryk University, Brno, Czechia

**Keywords:** REDs, relative energy deficiency in sport, athletes, markers, low energy availability

## Abstract

**Introduction:**

The review aims to summarize the markers used in diagnosing relative energy deficiency in sport (REDs) and compare them with the REDs CAT2 score.

**Methods:**

A systematic search was performed in the PubMed, Web of Science, and SPORTDiscus databases during April 2023. The descriptors used were “athlete” AND “REDs,” along with respective entry terms. The selection process followed the PRISMA 2020 recommendations, identifying 593 records, from which 13 studies were ultimately selected. Seventy-nine markers were identified and categorized into six groups: bone mineral density (BMD), metabolic resting rate, blood biomarkers, anthropometrics, nutritional intake, and performance parameters. The most frequently utilized biomarkers included BMD, anthropometric parameters (e.g., body mass index, body mass, and fat mass), and the triiodothyronine (T3) concentration.

**Results:**

According to the REDs CAT2 pointed indicators, the biomarkers varied among the studies, while 7 out of the 13 included studies achieved a ≥60% agreement rate with this tool. The prevalence of low energy availability, an etiological factor in the development of REDs, was detected in 4 out of 13 studies, with an average of 39.5%.

**Conclusion:**

In conclusion, this review highlights the most commonly used markers in diagnosing REDs, such as BMD, anthropometric parameters, and T3 hormone concentration. Due to the current inconsistencies, standardizing diagnostic methodologies is crucial for future research. By focusing on widely used markers, this review aids future research planning and result interpretation and points out the ongoing need for methodological consistency in evolving diagnostic tools.

**Systematic Review Registration:**

https://www.crd.york.ac.uk/, PROSPERO (CRD42022320007).

## Introduction

The phenomenon of energy deficiency in sports is a widespread problem among athletes and has emerged as a new syndrome called relative energy deficiency in sport (REDs). In cooperation with the International Olympic Committee (IOC), the concept of REDs and its first official definition were introduced in 2014 (1). REDs is characterized by low energy availability (LEA), causing a profound impact on physiological functions within the organism. It includes, but is not limited to, areas such as abnormalities in metabolic function, menstrual cycle, bone health, immunity, protein synthesis, and cardiovascular health (1). The first symptoms that drew attention to possible disturbances of the athlete's bodily functions were menstrual cycle abnormalities (2). Based on these observations, the female athlete triad (FAT) was created in 1992. The first version of FAT included amenorrhea, osteoporosis, and disordered eating (3). During ongoing research, the definition was updated to include (1) low energy availability with or without disordered eating, (2) low bone mineral density (BMD), and (3) menstrual dysfunction (4). Thus, research had focused primarily on female athletes up to this point. However, it became evident that low energy availability affects many more human health and performance areas. Furthermore, it also affects male athletes (1, 5). Therefore, as mentioned above, the concept of REDs was developed (1). Since 2014, studies have increasingly focused on male athletes, but the number of studies involving female athletes is still noticeably higher.

Although REDs has been widely accepted and respected among the sports science community, there are still numerous limitations in its practical application in monitoring athletes (6, 7).

The etiological factor for REDs is LEA (1, 8); therefore, the diagnosis needs to involve parameters related to LEA. The common practice is to use screening questionnaire tools, which are well applicable to the field and suited for the initial detection of at-risk athletes in large populations (9). Nonetheless, questionnaire tools should be cautiously evaluated due to the frequent design of self-reported questions. It is recommended that questionnaires be used along with objective, practical measurements to provide a more in-depth assessment (10). However, one of the biggest challenges is the unification of the diagnostic methods for REDs (11) and the different methodologies used in studies, which can lead to challenges in assessing and evaluating the research findings (10, 12). Significant progress in this area has been enabled by the latest 2023 IOC Consensus statement and the associated IOC REDs Clinical Assessment Tool-Version 2 (IOC REDs CAT2) (13, 14). This tool has undergone internal expert voting statement validation and external validation through cross-agreement among REDs experts in clinical settings, enabling the identification of a more refined set of markers suitable for diagnosing REDs (14).

Further challenges within the REDs field also involve identifying markers suitable for diagnosis, determining their cutoff values, and fostering more effective collaboration among experts. Despite the great importance of the IOC 2023 Consensus statement (13) and the IOC REDs CAT2 (14), their integration into the diagnostic process and research may require time. Therefore, it is still relevant to highlight the methodological inconsistencies present in current studies.

A comprehensive summary of the markers used to assess REDs in the existing literature is not yet available. Such a review, combined with insights from the REDs CAT2 tool, could assist in selecting a more specific set of markers to increase consistency across studies and facilitate the interpretation and comparison of results. Therefore, this review aims to bridge this gap by providing an overview of practical measurement methods, the frequency of their use in the included studies, and a comparison with the REDs CAT2 tool.

## Materials and methods

A systematic review was performed under the Preferred Reporting Items for Systematic Reviews and Meta-Analyses (PRISMA) guidelines, updated in 2020 (15), to answer the research question. The review was registered at PROSPERO with number CRD42022320007.

### Eligibility criteria and search strategy

Studies were eligible for inclusion if they met the following criteria: *participants:* athletes of both sexes, all disciplines, and advanced or elite level; *outcomes*: evaluation of the type, variety, number, and frequency of individual markers used in REDs diagnosis, as well as compliance with the IOC recommendation. Studies were ineligible if the outcomes of interest were not measured or the results were not described. Literature reviews, guidelines, letters to the editor, conference abstracts, dissertation thesis, and non-English language articles were also excluded. Considering that REDs was officially defined in 2014, the search was performed with a data range from March 2014 to. The search was conducted on Medline (via PubMed), Web of Science, and SPORTDiscus (via EBSCOhost) in April 2023. The search terms followed the descriptors from categories #1 and #2 related to “athlete” AND “relative energy deficiency” using the entry terms and derivative words (available in the Table 1).

**Table 1:** Characteristics of the included studies and categories of used markers (*n* = 13 studies).

	Sample characteristics		BMD	RMR	Blood biomarkers	Anthropometrics parameters	Nutritional intake	Performance
Study	Sex sport modality sample size/age	Control group	** **	✓	✓	✓	✓	✓
Hooper et al. (16)	NCAA Division 1 female distance runners *N* = 7/22.3 ± 1.5 years	—	—	—	✓	✓	✓	✓
Õnnik et al. (17)	High-level male and female *N* = 30/28.0 ± 3.75 years and *N* = 26/28.6 ± 6.34 years	Male and female control groups *N* = 29/24.1 ± 3.83 years and *N* = 29/24.97 ± 5.74 years	✓	✓	✓	✓	✓	✓
Torstveit et al. (18)	Well-trained male endurance athletes *N* = 53/35.3 ± 8.3 years	—	—	✓	✓	—	✓	✓
Keay et al. (19)	Competitive male road cyclists *N* = 45/36.2 ± 14.3 years	—	✓	✓	✓	✓	✓	✓
Stenqvist et al. (20)	Well-trained male cyclists *N* = 20/33.3 ± 6.7 years	—	✓	—	✓	✓^a^	—	✓
Keay et al. (21)	Competitive male road cyclists *N* = 50/35.0 ± 14.2	—	✓^a^	✓	✓	✓	—	✓
Stenqvist et al. (22)	Olympic-level male athletes *N* = 44/24.7 ± 3.8 years	–	✓	✓	–	✓	✓	✓
Mathisen et al. (23)	Female fitness athletes *N* = 25/28.1± 5.5 years	Female references *N* = 26/29.8± 6 years	✓	✓	✓	✓	✓	✓
Civil et al. (24)	Royal Conservatoire of Scotland female ballerinas *N* = 20/18.1 ± 1.1 years	—	✓	✓	✓	✓	✓	✓
Lee et al. (25)	Male Korean collegiate soccer players *N* = 10/9.1 ± 0.6 years	—	✓	—	✓^a^	✓	✓	—
Pritchett et al. (26)	National-level para-athletes: males and females *N* = 9/27 ± 8 years and *N* = 9/27 ± 7 years	—	✓	—	✓	✓	✓	—
Gibson-Smith et al. (27)	Elite climbers: males and females *N* = 20/29.1 ± 5.4 and *N* = 20/31.4 ± 7.7 years	—	—	✓	✓	✓	✓	✓
Kalpana et al. (28)	National-level male Kho-Kho players *N* = 52/16–31 years	—	✓	—	—	—	—	—

BMD, bone mineral density; T3, triiodothyronine; BMI, body mass index; BM, body mass; FM, fat mass; RMR, resting metabolic rate; EA, energy availability; EI, energy intake; FFM, fat-free mass; IGF-1, insulin-like growth factor 1; EEE, exercise energy expenditure; FTP, functional threshold power; GH, growth hormone, ALP, alkaline phosphatase; LBM, lean body mass; TEE, total energy expenditure; NEAT, non-exercise activity thermogenesis; DIT, dietary induced thermogenesis; TSH, thyroid-stimulating hormone; SHBG, sex hormone-binding globulin; FSH, follicle-stimulating hormone; LH, luteinizing hormone; WBC, white blood cell; RBC, red blood cell; SGOT, serum glutamic oxaloacetic transaminase; SGPT, serum glutamate pyruvate transaminase; LDL, low density lipoprotein; WHR, waist-to-hip ratio; VAT, visceral adipose tissue; AEE, activity energy expenditure; PR, personal record; IAFF score, International Association of Athletics Federations score.

^a^ These markers were evaluated via comparing groups with low vs. adequate energy availability. However, these conditions were only assessed using the questionnaire tools; therefore, these conclusions should be taken with caution.

### Selection process and data extraction

The articles were imported into Rayyan systematic review software to proceed with the selection process. This process was performed as follows: (1) a researcher (KD) uploaded the articles from each database and then (2) excluded the review articles, letters to the editor, duplicates, and articles in non-English languages (identified by the software); (3) two independent researchers (DP and AW) screened the articles’ titles and abstracts, and a third checked those excluded (KD); and (4) finally, two independent researchers (KD and AW) screened the full text of the articles for final inclusion. Any disagreements between reviewers were resolved by a third reviewer (AP). A prior pilot selection, with the first 25 articles, was performed to test the researchers’ understanding, demonstrating an agreement of 88% between the two reviewers (DP and AW). 

Data related to the sample characteristics (e.g., sex, sport modality, age, and size), the presence of REDs, biomarkers used in REDs diagnosis [e.g., hormones, resting metabolic rate, bone mineral density, blood glucose, body mass index (BMI), and cholesterol], and any potentially relevant outcomes were extracted from included studies by two researchers (KD and AW).

### Methodological quality

The assessment of methodological quality for the articles with a descriptive approach was performed using the STROBE tool (29) and for those with an intervention approach was performed by ROBINS-I (30). Three researchers participated in this phase (KD, AW, and AP). The STROBE checklist assesses the quality of cohort, case–control, and cross-sectional studies. It contains 22 items assessing risk factors for bias. Response options are a score of 0 if the articular checklist item is not fulfilled, 1 if the articular checklist item is fulfilled, and NA if the checklist item does not apply to the specific publication. Based on the sum of the total score and the percentage gain of the possible maximum, the quality of the study is then evaluated as follows: ≥85 = excellent, 70 to <85 = good, 50 to <70 = fair, and <50 = poor, as used previously. The ROBINS-I rating system is based on seven domains, each consisting of a subset of questions focusing on possible areas of systematic error. The domains include confounding, participants, classification of interventions, deviations from intended interventions, missing data, measurement of outcomes, and selection of the reported results. In this review, we used only domains 2–7 for evaluation; more details on this process are provided in the Discussion section. The response options are “Yes,” “Probably yes,” “Probably no,” “No,” and “No information.” Based on the continuous responses, each domain is then evaluated as a whole, and the rating of all the domains is reflected in the labeling of the study as “Low risk,” “Moderate risk,” “Serious risk,” and “Critical risk” of bias.

### REDs CAT2 agreement

The biomarkers used in the included studies were compared with the IOC REDs CAT2 (14), an improved version derived from the original IOC REDs Clinical Assessment Tool (CAT) introduced in 2015 (31). The development of the IOC REDs CAT2 involved internal validation through expert voting statements and external validation via clinical cross-agreement assessments by experts. The assessment protocol of IOC REDs CAT2 comprises three sequential steps:

I. Initial screening using population-specific REDs questionnaires or clinical interviews, with individuals deemed at risk moving on.
II. Assessment of various REDs signs/symptoms to uniform the Severity/Risk Assessment Tool and Stratification, with guidelines for sports participation; data obtained from these steps serve as the basis.
III. Physician-led final clinical diagnosis/stratification and associated implementation of a treatment plan, ideally involving a collaboration of a multidisciplinary health team and REDs performance (14).

Based on the scoring outcomes of primary and secondary indicators, the risk is categorized into four-color traffic-light severity/risk classifications, ranging from “none” to “very low,” “mild,” “moderate to high,” to “very high/extreme.” Recommendations concerning the monitoring of athletes, participation in training and competitions, and medical interventions complement these classifications. In addition, REDs CAT2 incorporates a set of potential indicators deemed emerging (14).

In the review process, markers identified in the included studies were compared to those outlined in the REDs CAT2. Given the focus on objective measurement methods, subjective markers obtained through interviews or questionnaires were omitted from this comparison. Subsequently, reviewer KD computed agreement rates between each study and the REDs CAT2 tool for scored, potential, and overall indicators. A second independent reviewer (AW) checked this process to ensure reliability.

## Results

### Study characteristics and methodological quality

**Figure 1:**
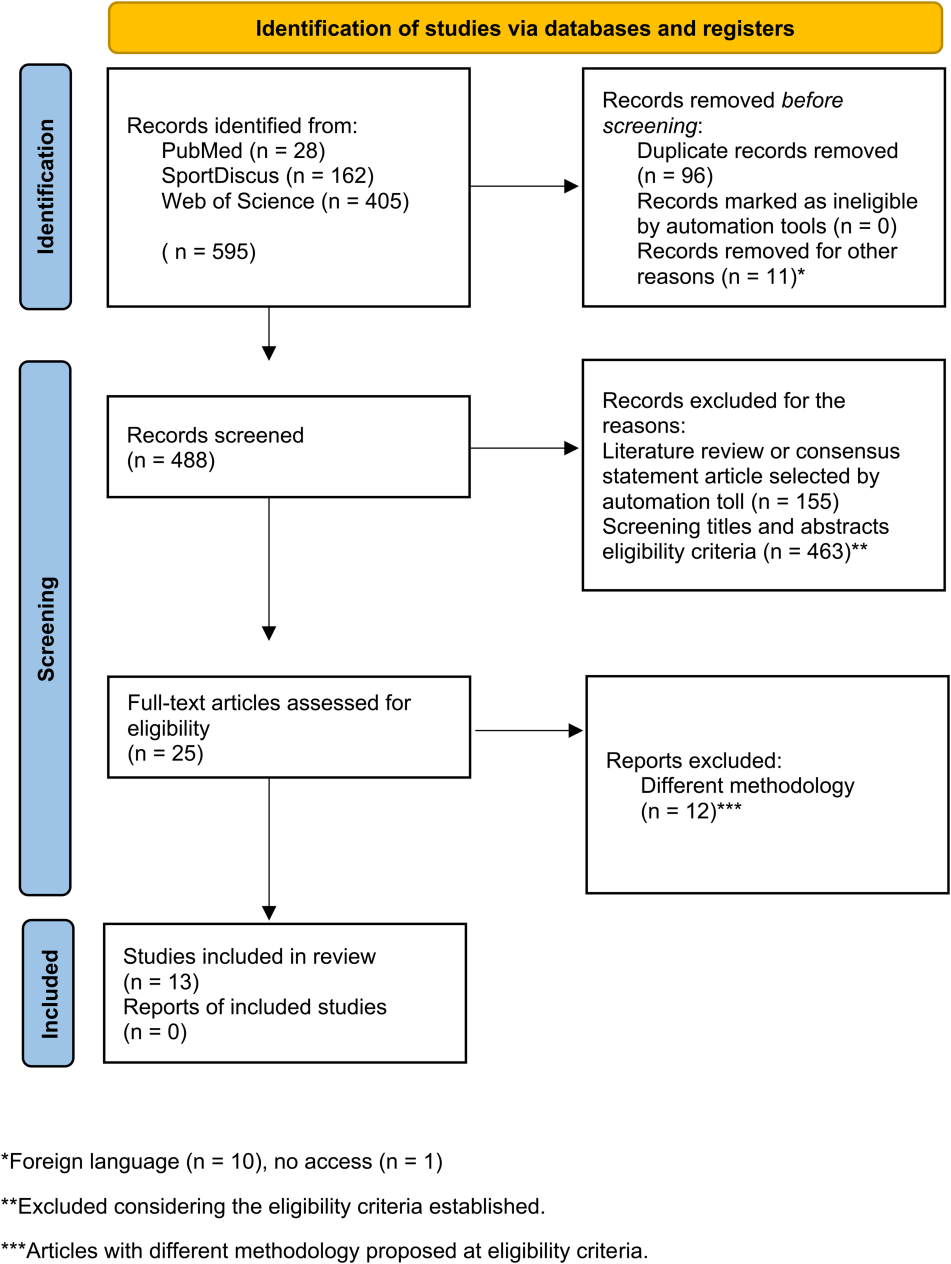
Flowchart diagram of the study selection process (PRISMA 2020) (15).

In total, 595 articles were found in the databases matching the combination of keywords entered. After excluding articles that were duplicates (*n* = 96) and for other reasons, such as those written in a foreign language (non-English) (*n* = 10) and with no access (*n* = 1), 488 articles were evaluated during the title and abstract screening. Of these articles, 155 were excluded through the review method, and 463 did not meet the eligibility criteria. For 25 articles, the full text was assessed; of these, 12 studies were excluded due to non-compatibility. Therefore, 13 studies were included in the final process (Figure 1).

The main characteristics and the categories of the REDs markers used in the included studies (bone mineral density, resting metabolic rate, blood markers, anthropometric parameters, nutritional intake, and performance) are presented in Table 1. Most of these studies focus on female athletes (7 out of 13); the most investigated disciplines were endurance sports, team sports, ballet, climbing, or a mix of disciplines or para-athletics disciplines. Athletes competed at the performance levels of well-trained, competitive, elite, national, and Olympic levels. Two studies also included a control group.

Among the 13 papers, 12 presented a descriptive study design and 1 presented an intervention design. For the descriptive ones, the methodological quality, assessed by the STROBE tool, demonstrated a range from good to excellent quality. Specifically, six studies were rated as excellent (17, 18, 20, 23–25) and six were rated as good (16, 21, 22, 26–28) (see the Table 2). One paper was designed as an intervention and demonstrated a moderated risk of bias based on the ROBINS-I tool.

**Table 2:** Overview of biomarkers used in REDs diagnosis and main outcomes (*n* = 13).

	REDs markers					
Study	BMD	RMR	Blood biomarkers	Anthropometric parameters	Nutritional intake	Performance
Hooper et al. (16)	—	↔ (pre-XC vs. post-XC) ↔ RMR ratio (any time point) ↑ (post-XC vs. pre-track) ↑ (pre-XC vs. pre-track)	T3 ↔ (any time point) Ferritin ↓ (pre-XC vs. post-XC) Ferritin ↑ (post-XC vs. pre-track) Vitamin D ↓ (pre-XC vs. post-XC) Vitamin D ↑ (post-XC vs. pre-track)	BMI ↔ (pre-XC vs. post-XC) BM ↔ (pre-XC vs. post-XC) BM ↑ (post-XC vs. pre-track) FFM ↔ (pre-XC vs. post-XC) FM ↔ (pre-XC vs. post-XC)	EA ↓ (vs. ACSM recommendations)	Performance relative to the PR ↔
Õnnik et al. (17)	LS-BMD ↔ (males, females) RF-BMD ↔ (males, females) TB-BMD ↔ (males, females)	—	LH ↔ FSH ↔prolactin ↔ testosterone ↔free T4 ↔ TSH ↔ T3 ↔GH ↔ IGF-1 ↔insulin ↓ (females) cortisol ↑ (males) WBC ↔ RBC ↓ (males) hemoglobin ↔ hematocrit ↓ (males) hematocrit ↔ (females) neutrophils ↔ (males) neutrophils ↑ (females) lymphocytes ↔ (males) lymphocytes ↓ (females) estradiol ↓ (males) estradiol ↔ (females) eosinophils ↔ basophils ↔	BMI males ↓ (vs. control) BMI females↓ (vs. control) BM females ↓ (vs. control) BM males ↓ (vs. control)	EI ↔ (males, females) relative EI (per kg body weight) ↑ (females) protein intake + relative value ↔ fat intake + relative value ↔ (males, females) carbohydrate intake + relative value ↔ (males) carbohydrate intake + relative value ↑ (females) dietary fiber intake ↔ dietary fiber intake relative value ↔ (males) dietary fiber intake relative value (per kg body weight) ↑ (females) sodium intake + relative value ↔ calcium intake ↑ calcium intake relative value ↔ (males) calcium intake relative value ↑ potassium intake ↔ potassium intake relative value ↑	VO_2_ max ↔ (measured only in athletes) IAFF score ↔ (points) (measured only in athletes)
Torstveit et al. (18)	—	RMR ↔ RMR ratio ↔ low RMR ratio ↔	Cortisol ↑ (group high EXDS score vs. group low) Cortisol highest quartile of range ↑ (group high EXDS score vs. group low) high cortisol (number of subjects) ↔ testosterone ↔ T3 ↔ IGF-1 ↔ insulin ↔ glucose ↔ testosterone:cortisol ratio ↔ cortisol:insulin ratio ↔	BMI ↔ BM ↔ FFM ↔ FM ↔ sleeping heart rate ↔	EEE (kcal/day) ↑ (group high EXDS score vs. group low) EI ↔ carbohydrate intake ↔ protein intake ↔ fat intake ↔ fiber intake ↔ energy balance (kcal/day) ↓ (group high EXDS score vs. group low) EA ↔ low EA (number of subjects with low EA) ↔	VO_2_ peak ↔ Active in sport ↔ exercise (hours/week) ↑ (group high EXDS score vs. group low)
Keay et al. (19)	↓ (Negative changes in both areas vs. before intervention) ↓ (negative changes in one areas vs. before intervention) ↑ (positive changes in both areas vs. before intervention) ↑ (positive changes in one areas vs. before intervention)	↓ (respondents with low EA) ↓ (respondents without skeletal loading exercise)	Testosterone ↔ testosterone/Z-score ↔ vitamin D ↑ (educated group vs. control group) vitamin D/Z-score ↔ T3 ↑ (educated group vs. control group) T3/Z-score ↔ albumin ↑ (educated group vs. control group) albumin/Z-score ↔ calcium ↔ alkaline phosphatase ↔ alkaline phosphatase/Z-score ↔ corrected calcium ↔ corrected calcium/Z-score ↔	—	EA ↑ (educated vs. control group)	Points gained over the racing season ↓ (group with negative changes in EA vs. before intervention) FTP ↓ (group with negative changes in EA vs. before intervention) points gained over the racing season ↑ (group with positive changes in EA vs. before intervention) FTP ↑ (group with positive changes in EA vs. before intervention)
Stenqvist et al. (20)	↔	Absolute RMR ↓ relative RMR ↓ RMR ratio ↓	Total testosterone ↑ free testosterone ↔ SHBG ↔ T3 ↓ cortisol ↑ insulin ↔ IGF-1 ↔ free testosterone:cortisol ratio ↔ total testosterone:cortisol ratio ↔	BMI ↔ BM ↔ FFM ↔ FM ↔	EI ↔ carbohydrate intake ↔ relative carbohydrate intake ↔ protein intake ↔ relative protein intake ↔ fat intake ↔ relative fat intake ↔	VO_2_ peak ↔ FTP (W) ↑ FTP (W/kg) ↑ aerobic peak power output (W) ↑ training volume per week ↔
Keay et al. (21)	↓^a^	—	Mean total testosterone ↓ (lower end of the reference range) mean vitamin D ↓ T3 ↔ (lower half of the reference range) albumin ↔ calcium ↔ alkaline phosphatase ↔	BMI^a^ ↓ FM^a^ ↓ VAT mass^a^ ↓	—	FTP (W/kg) ↔ training load ↔
Stenqvist et al. (22)	L1–L4 Z-score ↔ Femur Z-score ↔	RMR ratio ↓ (low vs. normal RMR) relative RMR ↓ (low vs. normal RMR)	Testosterone ↔ free testosterone ↔ T3 ↔ cortisol ↔ total cholesterol ↔ LDL cholesterol ↔	BMI ↔ BM ↔ FFM ↔ FFM index ↔ FM ↔ FM index ↔	—	Training volume per month ↔
Mathisen et al. (23)	↔	RMR FA ↓ (baseline vs. 2 weeks before competition)	—	FM ↓ (FA vs. FR) BMI ↔ BM ↔ LBM ↔ adult BM difference ↔ history of ED (self-reported) ↔ current ED (self-reported) ↔	EI (kcal) ↑ (FA vs. FR) EI (kcal/kg LBM) ↑ (FA vs. FR) carbohydrate intake (g) ↑ (FA vs. FR) carbohydrate intake (g/kg BW) ↑ (FA vs. FR) protein intake (g/) ↑ (FA vs. FR) protein intake (g/kg BW) ↑ (FA vs. FR) fat (energy %) ↑ (FA vs. FR) dietary fiber ↔	Experience with regular exercise ≥5 years ↔ exercising ≥5 times per week current year ↔
Civil et al. (24)	Total BMD ↔ Z-score ↔	↔	Vitamin D ↔	BM ↓ (after week of observation) BMI ↔ WHR ↔ FM ↔ FFM ↔	EI ↔ DIT ↔ EA (calculated) ↔ TEE (total energy expenditure) ↑ energy balance ↓ NEAT ↑ EEE ↑ fiber intake ↑ fluid intake ↑ fat intake ↔ carbohydrate intake ↔ protein intake ↔	Training volume per week (self-reported) ↔
Lee et al. (25)	BMD ↔ Z-score ↔	REE ratio ↓ REEm/FFM ↓	T3 ↔ cortisol ↔ insulin ↔ GH ↔ IGF-1 ↑ (vs. REE ratio) testosterone ↔ leptin ↔	BM↔ BMI ↔ FM ↔ FFM ↔ sleeping energy expenditure ↔	EI ↓ DIT ↓ EEE ↔ EPOC ↔ NEAT ↔ hourly resting energy expenditure ↔ TEE ↔ 24 h energy balance ↔ 24 h EA ↔ within-day energy balance <0 kcal (h/day) ↔ within-day energy balance <−400 kcal (h/day) ↔ largest hourly deficit (kcal) ↔	VO_2_ max ↔
Pritchett et al. (26)	Z-score ↓ (females) Z-score ↓ (males)	—	Testosterone ↓ (males) IGF-1^a^ ↑ (females) progesterone ↓ T3 ↔ estradiol ↔	—	—	—
Gibson-Smith et al. (27)	—	—	Serum ferritin ↓ (females)transferrin saturation ↔ sum of 8 SF (serum ferritin) ↑	BM ↓ BMI ↓ FM ↑ arm girth ↓ waist girth ↓ calf girth ↓ gluteal girth ↔	EI (kcal·kgFFM-1·day-1) ↑ (females) carbohydrate intake ↔ protein intake ↔ fat intake ↔ iron intake ↔ iron intake density (mg/1,000 kcal) ↔	—
Kalpana et al. (28)	Z-score ↓ bone mineral content ↔ BMD ↔ T-score ↔	BMR ↓	Serum calcium ↔ serum vitamin D3 ↔ serum free T3 ↔ hemoglobin ↔ serum albumin ↔ serum creatine ↔ SGOT ↔ SGPT ↔	BM ↔ FM ↔ overall sleep quality ↓ LBM ↑	EA ↓ carbohydrate intake ↓ protein intake ↓ fat intake ↓ vitamin A intake ↓ vitamin B2, B6, B9 intake ↓ iron intake ↓ zinc intake ↓ fluid intake ↓ AEE ↑ daily energy expenditure ↑ EI ↓ daily energy expenditure/BMR ↔	Agility ↓ speed ↔

Pre-CX, athletes before cross-country season; Post-CX, athletes after cross-country season; Pre-track, athletes before track season; low RMR ratio, number of subjects with low RMR; BMD, bone mineral density; T3, triiodothyronine; T4, thyroxine; BMI, body mass index; BM, body mass; FM, fat mass; EXDS score, exercise dependence scale score; RMR, resting metabolic rate; EA, energy availability; EI, energy intake; FFM, fat-free mass; IGF-1, insulin-like growth factor 1; EEE, exercise energy expenditure; FTP, functional threshold power; GH, growth hormone, ALP, alkaline phosphatase; LBM, lean body mass; TEE, total energy expenditure; NEAT, non-exercise activity thermogenesis; DIT, dietary induced thermogenesis; TSH, thyroid-stimulating hormone; SHBG, sex hormone-binding globulin; FSH, follicle-stimulating hormone; LH, luteinizing hormone; WBC, white blood cells; RBC, red blood cells; SGOT, serum glutamic oxaloacetic transaminase; SGPT, serum glutamate pyruvate transaminase; LDL, low density lipoprotein; WHR, waist-to-hip ratio; VAT, visceral adipose tissue; AEE, activity energy expenditure; PR, personal record; IAFF score, international association of athletics federations score.

Signs ↑ (increase) and ↓ (decrease) indicate a statistically significant result, and sign ↔ indicates a statistically insignificant result.

^a^ The markers were evaluated via comparing groups with low vs. adequate energy availability.

### Overview of biomarkers used in REDs diagnosis and the frequency of their use

We found 79 biomarkers used to determine the presence of REDs in the 13 included studies. Table 2 presents the complexity and diversity of the biomarkers used within the included studies. The biomarkers were categorized into five groups (bone mineral density biomarkers, resting metabolic rate biomarkers, blood biomarkers, anthropometric parameters, nutritional intake parameters, and performance parameters). All 13 studies used at least two (or more) categories of markers to determine the presence of REDs.

Table 3 presents the quantification of biomarkers used to assess REDs and complements Table 2. It shows a comprehensive overview of the frequency of their use in the included studies. The biomarkers most often used were BMD, BMI, BM (body mass), FM (fat mass), and T3 (triiodothyronine) blood concentrations, which were involved in 10 of the 13 studies (76.9%). Nine studies (69.2%) used the measurement of RMR (resting metabolic rate), while 8 studies (61.5%) used total testosterone level and EI (energy intake). Seven studies (53.9%) used nutritional parameters such as carbohydrate, protein, and fat intake EA (energy availability) was used in six studies (46.2%).

**Table 3:** Frequency of markers measured among the studies.

Number of studies	Relative frequency	Markers
10	76.9	BMD, T3, BMI, BM, FM
9	69.2	RMR
8	61.5	Total testosterone, EI
7	53.9	Carbohydrate intake, protein intake, fat intake
6	46.2	EA, FFM, training volume
5	38.5	Vitamin D, cortisol, IGF-1
4	30.8	Insulin, dietary fiber intake, EEE
3	23.1	Albumin, calcium, energy balance, FTP, TEE
2	15.4	Ferritin, free testosterone, estradiol, GH, hemoglobin, ALP, LBM, iron intake, fluid intake, NEAT, DIT, VO_2_max, VO2 peak
1	7.7	Free thyroxine, transferrin saturation, SHBG, prolactin, LH, FSH, progesterone, TSH, leptin, glucose, WBCs, RBCs, hematocrit, neutrophils, lymphocytes, basophils, creatine, SGOT, SGPT, total cholesterol, LDL, WHR, girth measurement, VAT, vitamin A intake, vitamin B2 intake, vitamin B6 intake, vitamin B9 intake, calcium intake, sodium intake, potassium intake, zinc intake, AEE, sleeping heart rate, overall sleep quality, sleeping energy expenditure, points gained over the racing season, performance relative to the PR, agility, IAFF score, speed, aerobic peak power

BMD, bone mineral density; T3, triiodothyronine; BMI, body mass index; BM, body mass; FM, fat mass; RMR, resting metabolic rate; EA, energy availability; EI, energy intake; FFM, fat-free mass; IGF-1, insulin-like growth factor 1; EEE, exercise energy expenditure; FTP, functional threshold power; GH, growth hormone, ALP, alkaline phosphatase; LBM, lean body mass; TEE, total energy expenditure; NEAT, non-exercise activity thermogenesis; DIT, dietary induced thermogenesis; TSH, thyroid-stimulating hormone; SHBG, sex hormone-binding globulin; FSH, follicle-stimulating hormone; LH, luteinizing hormone; WBCs, white blood cells; RBCs, red blood cells; SGOT, serum glutamic oxaloacetic transaminase; SGPT, serum glutamate pyruvate transaminase; LDL, low density lipoprotein; WHR, waist-to-hip ratio; VAT, visceral adipose tissue; AEE, activity energy expenditure; PR, personal record; IAFF score, international association of athletics federations score.

## Discussion

This review systematically compiles a list of methods utilized in diagnosing REDs. Our analysis of included studies revealed that the most frequently used biomarkers in current studies are BMD, BMI, BM, FM, and blood T3 concentration, included in 10 out of 13 studies (76.9%).

While the 2023 IOC Consensus statement marked a significant milestone in selecting appropriate diagnostic markers for REDs, the authors emphasized the necessity for ongoing updates and revisions. This included refining the range of recognized *sequelae* associated with REDs and reassessing the markers themselves. Thus, a critical examination of the strengths and limitations of these markers, alongside evaluating their ability to reflect individuals’ health status accurately, remains imperative.

### Anthropometric parameters

Anthropometric parameters, such as BMI and body composition, are widely used in medical practice. According to the Centers for Disease Control and Prevention's recommendations for general practitioners (32), BMI is a simple, inexpensive, and non-invasive method of estimating body fat and health risk, requiring no special equipment. However, several studies have pointed to the inaccuracy of BMI, particularly among patients with different ethnic backgrounds or an inability to distinguish body weight between body fat and muscle mass (33, 34). Thus, although this calculation can provide valuable information in the REDs diagnostic process, as with other markers, it cannot be evaluated in isolation (35). According to REDs CAT2, BMI is considered a potential indicator in assessing REDs risk, underscoring that the need for further research to quantify the parameters and cutoffs more accurately (14).

To accurately determine body composition, it is necessary to use valid methods that contribute to an objective assessment of the athlete’s overall condition. Body composition and adipose tissue thicknesses can be accessed via skinfold measurement. However, B-mode ultrasound is a more reliable and preferred method, which can provide good results even in lean individuals (36). Despite its costliness, the dual-energy x-ray absorptiometry (DXA) measurement is also the recommended method of choice as the gold standard for assessing body composition (37).

The authors of the IOC Consensus statement also pointed out that too much focus on anthropometric parameters and body composition can intensify the pressure placed on athletes, especially on adolescents under the age of 18 years (13, 38). It is, therefore, essential to identify valid and reliable methods and develop guidelines for interpreting, managing, and communicating with athletes (39).

### Bone health

Biomarkers assessing bone health are among the most used, as shown by the results of this review. Impaired bone health has been associated with low energy availability from its onset. It was also included in the original definition of the female athlete triad (34), from which the concept of REDs was developed (1). Low energy availability affects bone health through reduced levels of hormones such as estrogen, leptin, and T3 associated with insulin-like growth factor 1 (IGF-1) secretion (40–43). In addition, inadequate intake of essential nutrients, including protein, calcium, or vitamin D, has been linked to the low energy intake associated with REDs (44, 45).

DXA is the most used method for measuring bone mineral density (46) and is also noted as a “preferred method” in the 2023 IOC Consensus statement (13). According to REDs CAT2, the authors recommend the following as a positive finding:

• *Premenopausal women and men aged <50 years: BMD Z-score <−1 at the lumbar spine, total hip, or femoral neck or decreased BMD Z-score from previous testing*.
• *Children/adolescents: BMD Z-score <−1 at the lumbar spine or total body less head or decreased BMD Z-score from the last testing (may be due to bone loss or insufficient bone gain)* (14).

Some previously published studies on energy availability have also used markers of bone turnover derived from blood samples. The research findings by Ihle and Loucks (47) suggest that changes may be apparent after 3 days of LEA. The findings of the study by Papageorgiou et al. (48) showed that 5 days of LEA below 15 kcal/day leads to changes in bone turnover markers in women, but no significant changes were found in men. A year later, Papageorgiou et al. (49) conducted another study involving a group of eumenorrheic women in whom 3-day LEA through dietary energy restriction resulted in changes in bone formation but not bone resorption. However, these bone turnover markers are not established due to the number of factors that may influence them. The time taken for the manifestation of changes might also be significantly influenced by variables such as the severity of LEA.

Moreover, markers reporting bone mineral density status should continually be assessed in the context of supplementary information, considering the specificity of each sport discipline. For example, the bone density of weightlifters generally reaches higher values than the reference range (50), and average values may indicate reduced BMD in these athletes.

### Resting metabolic rate

RMR was assessed in nine of the 13 included studies, accounting for 69.2% (see Table 3). RMR represents the energy necessary to maintain homeostasis while at rest. Unlike basal metabolic rate, which necessitates strict conditions such as a 12-h fasting period and a thermoneutral environment, RMR can be measured throughout the day (51). The suppressed RMR associated with LEA may be explained by adaptive responses aimed at conserving energy (52).

Various methodologies are employed in studies to determine RMR. Indirect calorimetry is often called the gold standard but requires specialized equipment (53). Consequently, researchers usually resort to estimating RMR using predictive equations, such as those proposed by Cunningham (54), Harris and Benedict (55), or Owen et al. (56). Another approach is the RMR ratio, defined as the ratio between measured RMR and predicted RMR. Some studies suggest that the RMR ratio serves as a valid indicator of LEA (57, 58). However, it is advisable to evaluate RMR in conjunction with other markers due to variations in the degree of metabolic suppression among athletes. These variations are influenced by factors such as the severity of LEA (58).

The 2023 IOC Consensus statement recognizes RMR testing as a “used and recommended” method for identifying impaired energy metabolism. Specifically, the endorsed procedures include indirect or room calorimetry measurements (13). In addition, REDs CAT2 identifies RMR as a potential indicator, with a reduced or low RMR [<30 kcal/kg fat-free mass (FFM)/day] or an RMR ratio (<0.90) considered indicative of the condition (14). However, Sterringer and Larson-Meyer (59) pointed out that a threshold of 0.9 may not be appropriate for all cases. In particular, for studies using the Cunningham equation from 1991 (60) or DEXA measurement, a threshold of 0.9 may lead to an underestimation of the prevalence of LEA.

### Blood biomarkers—hormone concentration

One of the most used markers in the included studies (76.9%) was T3. It is one of the hormonal agents released by the thyroid gland and is indispensable in energy metabolism and growth (61). T3 is also involved in the reproductive process (62) and bone tissue metabolism through the local production of IGF-1 (63). Although its concentration is strongly associated with metabolic functions, this marker still needs to be evaluated in the context of other methods. This is because its concentration may be affected by many conditions, such as circadian rhythms, thyroid disease, alterations in serum binding proteins, or other associated medical conditions (64, 65). Clinically or subclinically low total or free T3 is also considered one of the primary REDs indicators listed in REDs CAT2 (14), and clinically or subclinically low IGF-1 is included in the list of potential indicators.

Testosterone concentration is also a frequently used marker in the studies (61, 5%). Subclinically low total or free testosterone is listed in REDs CAT2 primary indicators; clinically low total or free testosterone is considered a severe primary indicator (counted as two primary indicators) (14). While disturbances in the menstrual cycle may affect the hypothalamic–pituitary–gonadal axis in women, this condition may not be detected as early in male athletes. Thus, for male athletes, in addition to testosterone levels, it is often necessary to consider self-reported data, such as the presence of low libido or decreased frequency of morning erections, in the diagnosis of REDs. Thus, as already mentioned, a combination of diagnostic methods is required. In women, low energy availability disrupts luteinizing hormone (LH) pulsatility, which further affects the hypothalamic–pituitary–gonadal axis, including levels of follicle-stimulating hormone (FSH), estrogens, and progesterone (66, 67). Two studies tested estradiol levels (17, 26), while one study tested levels of LH, FSH (17), and progesterone (26). However, the REDs CAT2 tool does not directly use these hormones as female reproductive cycle function indicators. Instead, it uses self-reported data on the presence of primary amenorrhea, secondary amenorrhea, or oligomenorrhea (14). LEA also affects other endocrine pathways such as cortisol, leptin, growth hormone, IGF-1 axis, sympathetic and parasympathetic tone, or thyroid hormones (66).

### Calculation of energy availability

The calculation of energy availability has been used in 46.2% of the included studies (6 out of 13). Given that low energy availability is a direct etiological factor in developing REDs (1), its inclusion in diagnostic methods appears logical. The variables required for the calculation of energy availability can also be obtained in a non-invasive and non-burdensome way. The prevalence of low energy availability was detected in four of the thirteen included studies: 67% (16), 23% (18), 22% (24), and 46% (28). In another study, a prevalence of 28% was assessed through the SEAQ-I questionnaire (21). In conclusion, the mean observed prevalence of LEA across the studies is 39.5%.

However, previous studies have indicated that calculating energy availability carries a high risk of error (9). Sources of this inaccuracy can include energy intake, while data obtained through nutritional recall may underestimate actual intake by 10%–20% (68, 69), and even cases of an underestimation of 50% are not uncommon (70). The measurement of energy expenditure also needs to be evaluated cautiously. Various methods of assessing energy expenditure are used across studies, such as doubly labeled water technique, direct calorimetry, indirect calorimetry, accelerometry, heart rate monitoring, or pedometry (71). Nevertheless, using more accurate methods is often complicated by the high cost of these devices in research settings. Therefore, epidemiological studies frequently rely on self-reported methods, which can lead to significant inaccuracies in the observed outcomes (72). Thus, calculating energy availability may serve as a valuable complementary method for diagnosing REDs and could also be beneficial in determining the optimal therapeutic approach (73). However, like other markers, it should not be evaluated in isolation.

### Reference markers according to IOC REDs CAT2

Although there is still no uniform and standardized methodological approach for the diagnosis of REDs, the new IOC Consensus statement of 2023 provides a comprehensive overview of:

(I) Preferred methods;
(II) Used and recommended methods; and
(III) Potential methods applicable for these purposes (13).

The IOC REDs CAT2 is closely aligned with this document and summarizes the LEA indicators, including symptoms and signs, that have emerged as current best practices for clinical assessment and research. Based on the evaluation of these indicators, an athlete may be included in one of the four-color traffic-light severity/risk categories. Each category is also associated with recommendations for athletic participation, athlete monitoring, medical intervention, or even full medical support, which may require the athlete’s temporary exclusion from training and competition (14).

The authors of the REDs CAT2 emphasize that this advanced tool should not be used in isolation but in combination with clinical consideration and other tools, such as screening questionnaires. In addition, they warn that the tool's reliability decreases if all the included indicators cannot be assessed and that REDs CAT2 is not a substitute for professional clinical diagnosis, advice, and/or treatment (13). Nevertheless, REDs CAT2 represents a scientifically supported system for evaluating LEA indicators and was selected as a reference tool to assess the quality of the markers used in the included studies.

As this review primarily focuses on objective methods of practical measurement, some subjective indicators obtained through interviews or questionnaires were excluded from this comparison. However, as previously mentioned, objective and subjective methods cannot be entirely separated, and combining them is desirable. After excluding methods that are not objectively measurable, 15 markers were identified in the reference tool. Five of these markers are scored as primary or secondary indicators; 10 potential markers are not scored but are considered emerging. An overview of the included and excluded indicators and the results of the agreement can be found in Table 4. The highest agreement with the CAT2 REDs was achieved in the study of Stenqvist et al. (22) using 80% of the scored indicators. Six studies used 60% (19–21, 25, 26, 74), two used 40% (18, 28), three used 20% (16, 23, 24) of the scored indicators, while one study did not include any of these scored markers but only the potential ones (27).

**Table 4:** Agreement of used markers according to IOC REDs CAT2 (*n* = 13 studies).

REDs indicator (14)	Hooper et al. (16)	Õnnik et al. (74)	Torstveit et al. (18)	Keay et al. (19)	Stenqvist et al. (20)	Keay et al. (21)	Stenqvist et al. (22)	Mathisen et al. (23)	Civil et al. (24)	Lee et al. (25)	Pritchett et al. (26)	Gibson-Smith et al. (27)	Kalpana et al. (28)
Severe primary indicators (count as two primary indicators)													
Primary amenorrhea (females: primary amenorrhea is indicated when there has been a failure to menstruate by age 15 in the presence of normal secondary sexual development (two SDs above the mean of 13 years) or within 5 years after breast development if that occurs before age 10) or prolonged secondary amenorrhea (absence of 12 or more consecutive menstrual cycles) due to FHA^a^													
Clinically low free or total testosterone (males: below the reference range)^b^	—	Yes	Yes	Yes	Yes	Yes	Yes	—	—	Yes	Yes	—	—
Primary indicators													
Secondary amenorrhea (females: absence of 3–11 consecutive menstrual cycles) caused by FHA^a^													
Subclinically low total or free testosterone (males: within the lowest 25% (quartile) of the reference range)^b^	—	Yes	Yes	Yes	Yes	Yes	Yes	—	—	Yes	Yes	—	—
Subclinically or clinically low total or free T3 (within or below the lowest 25% (quartile) of the reference range)	Yes	Yes	Yes	Yes	Yes	Yes	Yes	—	—	Yes	Yes	—	Yes
History of ≥1 high-risk (femoral neck, sacrum, pelvis) or ≥2 low-risk BSI (all other BSI locations) within the previous 2 years or absence of ≥6 months from training due to BSI in the previous 2 years^a^													
Premenopausal females and males <50 years old: BMD Z-score^a^ <−1 at the lumbar spine, total hip or femoral neck or decrease in BMD Z-score from prior testing Children/adolescents: BMD Z-score^a^ <−1 at the lumbar spine or TBLH or decrease in BMD Z-score from prior testing (can occur from bone loss or inadequate bone accrual)	—	Yes	—	Yes	Yes	Yes	Yes	Yes	Yes	Yes	Yes	—	Yes
A negative deviation of a pediatric or adolescent athlete's previous growth trajectory (height and/or weight)	—	—	—	—	—	—	—	—	—	—	—	—	—
An elevated score for the EDE-Q global (>2.30 in females; >1.68 in males) and/or clinically diagnosed DSM-5-TR-defined eating disorder^a^ (only one primary indicator for either or both outcomes)^a^													
Secondary indicators													
Oligomenorrhea caused by FHA (>35 days between periods for a maximum of 8 periods/year)^a^													
History of 1 low-risk BSI (see high vs. low-risk definition above) within the previous 2 years and absence of <6 months from training due to BSI in the previous 2 years^a^													
Elevated total or LDL cholesterol (above reference range)	—	—	—	—	—	—	Yes	—	—	—	—	—	—
Clinically diagnosed depression and/or anxiety (only one secondary indicator for either or both outcomes)^a^													
Potential indicators (not scored, emerging)													
Subclinically or clinically low IGF-1 (within or below the lowest 25% (quartile) of the reference range)	—	Yes	Yes	—	Yes	—	—	—	—	Yes	Yes	—	—
Clinically low blood glucose (below the reference range)	—		Yes	—	—	—	—	—	—	—	—	—	—
Clinically low blood insulin (below the reference range)	—	Yes	Yes	—	Yes	—	—	—	—	Yes	—	—	—
Chronically poor or sudden decline in iron studies (e.g., ferritin, iron, transferrin) and/or hemoglobin	Yes	Yes	—	—	—	—	—	—	—	—	—	Yes	Yes
Lack of ovulation (via urinary ovulation detection)^a^													
Elevated resting AM or 24 h urine cortisol (above the reference range or significant change for an individual)	—	Yes	Yes	—	Yes	—	Yes	—	—	Yes	—	—	—
Urinary incontinence (females)^a^													
GI or liver dysfunction/adverse GI symptoms at rest and during exercise^a^													
Reduced or low RMR <30 kcal/kg FFM/day or RMR ratio <0.90	Yes	—	Yes	Yes	Yes	—	Yes	Yes	Yes	Yes	—	—	—
Reduced or low libido/sex drive (especially in males) and decreased morning erections^a^													
Symptomatic orthostatic hypotension	—	—	—	—	—	—	—	—	—	—	—	—	—
Bradycardia (HR <40 in adult athletes; HR <50 in adolescent athletes)	—	—	—	—	—	—	—	—	—	—	—	—	—
Low systolic or diastolic BP (<90/60 mm Hg)	—	—	—	—	—	—	—	—	—	—	—	—	—
Sleep disturbances	—	—	—	—	—	—	—	—	—	—	—	—	Yes
Psychological symptoms (e.g., increased stress, anxiety, mood changes, body dissatisfaction and/or body dysmorphia)^a^													
Psychology symptoms^a^													
Exercise dependence/addiction^a^													
Low BMI	Yes	Yes	Yes	—	Yes	Yes	Yes	Yes	Yes	Yes	—	Yes	—
Agreement													
Pointed indicators (*n* = 5)	20%	60%	40%	60%	60%	60%	80%	20%	20%	60%	60%	0%	40%
Potential indicators (*n* = 11)	27.3%	45.5%	54.5%	9.1%	45.5%	9.1%	27.3%	18.2%	18.2%	45.5%	9.1%	18.2%	18.2%
Overall	25%	50%	50%	25%	50%	25%	43.8%	18.8%	18.8%	50%	25%	12.5%	25%

BMD, bone mineral density; BMI, body mass index; BP, blood pressure; BSI, bone stress injuries; DSM-5-TR, diagnostic and statistical manual of mental disorders, fifth edition, text revision; DXA, dual-energy x-ray absorptiometry; EDE-Q, eating disorder examination questionnaire; FFM, fat-free mass; FHA, functional hypothalamic amenorrhea; GI, gastrointestinal; HR, heart rate; traffic-light severity/risk categories, insulin-like growth factor 1; ISCD, International Society for Clinical Densitometry; LDL, low-density lipoprotein; LSC, least significant change; RMR, resting metabolic rate; T3, triiodothyronine; T, testosterone; TBLH, total body less head.

^a^ Gray rows show indicators that cannot be objectively measured and that, therefore, were excluded for the purpose of marker agreement in this review.

^b^ Testosterone level, which is included in the “severe primary indicators” and “primary indicators” categories, was considered as one indicator (not counted twice) to calculate agreement in marker use.

### Limitations

The main limitation, not only of this review but to the entire field of REDs, is that no single marker or group of markers can reliably indicate the presence of REDs in athletes at this time. Therefore, we can only determine athletes’ risk levels as “low/moderate/high” rather than diagnosing the presence or absence of REDs. REDs cannot be diagnosed based on a single variable. Instead, several factors must be considered. Thus, this review can only provide an overview of the markers used in REDs diagnosis in current studies and highlight their frequency of use. The most commonly used markers were also analyzed with respect to the REDs CAT2 tool. Another potential source of error is the assessment of study quality and the risk of bias. Although three researchers performed these tasks independently, evaluating individual questions and the overall evaluation of the included categories might be influenced by subjective perceptions or interpretations of the questions related to the REDs topic.

### Future directions

The process of diagnosing REDs is currently fragmented, with studies employing various methods and a broad range of markers in their methodologies, as evidenced by the findings of this review. In addition, determining the presence or absence of REDs is challenging. In response, it is crucial to identify reliable markers suitable for diagnosing REDs, establish diagnostic cutoffs, and develop guidelines for their evaluation (13). It is essential to approach this condition holistically, considering factors that may influence the final diagnosis, such as the age of the athletes, their overall nutritional status, or the type and intensity of their training schedule. Furthermore, the importance of interdisciplinary and multidisciplinary collaboration in diagnosing, treating, and preventing this syndrome cannot be overstated, as it is necessary to improve the future approach to REDs. The fragmentation of complex conditions like REDs can lead to erroneous conclusions and flawed therapeutic strategies (75). The prevention of REDs should not rely solely on the sports physician. Coaches, physiotherapists, nutritional therapists, psychiatrists, the athletes themselves and, when appropriate, their parents should all be involved in every part of this process–primary, secondary, and tertiary REDs prevention (73, 76).

## Conclusion

This review is among the first articles to summarize the type and frequencies of markers used in REDs diagnosis in current studies. A focus on unifying the methodology for diagnosing REDs is essential for future research, as the variety of markers and inconsistent methodologies may complicate the interpretation of results. This review identified that the most commonly used markers were BMD, anthropometrical parameters (e.g., BMI, BM, and FM), and T3 hormone concentration (76.9% of the included studies). RMR (69.2% of the included studies), testosterone concentration, and energy intake calculation (61.5% of the included studies) also had a high frequency of use. According to the REDs CAT2 (14), the highest agreement was achieved in the study by Stenqvist et al. (22) using 80% of the scored indicators. Six studies used 60% (19–21, 25, 26, 74), two used 40% (18, 28), three used 20% (16, 23, 24) of the scored indicators, while one study did not include any of these scored markers, only the potential ones (27).

The calculation of energy availability, a direct etiological factor for developing REDs, was used in 46.2% of the included studies. Despite its simplicity and broad applicability, this marker has the disadvantage of a potentially significant risk of error in calculating energy intake and expenditure during physical activity. Thus, it should be evaluated in combination with other methods.

This summary of the markers used in REDs diagnosis may help future researchers focus on the most widely used markers when planning research and facilitate interpreting research results. Incorporating new tools into research and medical care will likely take some time. Therefore, it remains relevant to highlight the inconsistency of methods used in current studies.

## Data Availability

The original contributions presented in the study are included in the article/Supplementary Material, further inquiries can be directed to the corresponding author.
